# Protein metabolism and physical fitness are physiological determinants of body condition in Southern European carnivores

**DOI:** 10.1038/s41598-020-72761-6

**Published:** 2020-09-25

**Authors:** Nuno Santos, Mónia Nakamura, Helena Rio-Maior, Francisco Álvares, Jose Ángel Barasona, Luís Miguel Rosalino, Maria João Santos, Margarida Santos-Reis, Pablo Ferreras, Francisco Díaz-Ruiz, Pedro Monterroso

**Affiliations:** 1 CIBIO/InBIO, Research Center in Biodiversity and Genetic Resources, University of Porto, Vairão, Portugal; 2 Departamento de Biologia, Faculdade de Ciências, Universidade do Porto, Porto, Portugal; 3 Faculty of Veterinary, VISAVET Centre and Animal Health Department, Complutense University of Madrid, Madrid, Spain; 4 cE3c-Centre for Ecology, Evolution and Environmental Changes, Faculdade de Ciências da Universidade de Lisboa, Lisboa, Portugal; 5 University Research Priority Program in Global Change and Biodiversity and Department of Geography, University of Zurich, Zurich, Switzerland; 6 Instituto de Investigación en Recursos Cinegéticos (IREC, CSIC-UCLM-JCCM), Ciudad Real, Spain; 7 Dept. Biología Animal, Facultad de Ciencias, Biogeography, Diversity, and Conservation Research Team, Universidad de Málaga, Málaga, Spain

**Keywords:** Ecophysiology, Physiology, Ecology

## Abstract

The physiological significance of biometric body condition indices (bBCI) is poorly understood. We hypothesized that bBCI are composite metrics of nutritional physiology, physical fitness and health. To test this hypothesis, we first compared the performance of eight bBCI, using 434 Southern European carnivores from six species as a model system; and then identified, by non-destructive methods, the hematology and serum biochemistry correlates of three selected bBCI. Fulton’s K Index, Major Axis Regression Residuals and Scaled Mass Index were the only bBCI insensitive to the effect of sex and age. The most informative physiological parameters in explaining the variation of these bBCI were the albumin (Effect Size (ES) = − 1.66 to − 1.76), urea (ES = 1.61 to 1.85) and total bilirubin (ES = − 1.62 to − 1.79). Hemoglobin and globulins (positive) and cholesterol (negative) were moderately informative (0.9 <|ES|< 1.5). This study shows that most bBCI do not control for the effect of age and sex in Southern European carnivores. Our results support that bBCI are composite measures of physiologic processes, reflecting a positive gradient from protein-poor to protein-rich diets, accompanied by increased physical fitness. Biometric body condition indices allow the integration of ecologically relevant physiological aspects in an easily obtained metric.

## Introduction

Body condition, i.e., the relative capacity of an organism to maintain the optimal functionality of its vital systems, reflects a combination of individual nutritional and health state^1–3^. It is often understood as a proxy for individual Darwinian fitness or related traits, such as foraging success and reproduction^2–8^ and thus is of fundamental interest in ecology and conservation^1,2,5,9,10^. Nevertheless this relationship between body condition and Darwinian fitness remains controversial and poorly known^2,3,7^.

Biometric body condition indices (bBCI) are frequently used as measurements of body condition as they are easy to estimate from data obtained straightforwardly from live-trapped or dead animals^2,9,11^. Many different bBCI have been described based on the relation between body mass and some measure of structural body size^9,11,12^. A reliable bBCI should control for the different relationship between mass and body size across sex and age class^1,12^, however be sensitive to fluctuations in individual body condition, for example, following seasonal pulses in resource availability^13,14^.

Biometric BCIs have often been taken as proxies of lipid reserves^8,15^; however, this could be an oversimplification of the physiological conditions that result in variations in body condition, for several reasons. First, there is a high inconsistency across studies on the relationship between lipid reserves and body condition^8,15^, likely because Darwinian fitness costs increase at both low and high lipid reserve’s values^2,4,16^. Second, lean species, such as carnivores, tend to accumulate less lipid reserves compared to herbivores or omnivores^17,18^, and animals at lower latitudes tend to rely less on lipid deposits than those at higher latitudes, possibly due to the lower seasonality in resource availability and to the trade-offs with thermal regulation^19^. Third, many interrelated components of nutritional physiology, notably protein reserves, likely contribute to the overall body condition^2,8,13,20,21^. Fourth, besides nutrition, body condition depends on other physiological components, such as physical fitness, i.e. the ability to carry out daily tasks with vigor and alertness without undue fatigue and capacity to handle unforeseen emergencies^22^. Physical fitness affects mobility and directly influences resource acquisition rates^3,18^. Fifth, the health status of the organism is also likely to affect body condition. Health status is determined by an organism’s resistance and tolerance to pathogens, which are mediated by its immune response^9,23^. Assembling and maintaining an immune response is energetically demanding for the organism, reducing the availability of energy for other physiological activities such as reproduction^24,25^, making it a significant driver of body condition^26^.

Thus, we postulate that bBCI are composite metrics of body condition, encompassing nutritional physiology, physical fitness and health. We test this hypothesis on six species of Southern European carnivores, from five families – Canidae (red fox *Vulpes vulpes* and wolf *Canis lupus*), Herpestidae (Egyptian mongoose *Herpestes ichneumon*), Viverridae (common genet *Genetta genetta*), Mustelidae (stone marten *Martes foina*), and Felidae (wildcat *Felis silvestris*). More specifically we compare eight (Table 1) widely used body condition indices—Mass/Length Ratio, Body Mass Index, Fulton’s K Index, Relative Condition, Ordinary Least Squares Regression Residuals, Major Axis Regression Residuals, Reduced Major Axis Regression Residuals and Scaled Mass Index—to assess their compliance with the characteristics of an efficient bBCI, and determine their relationship with hematology and serum biochemistry.

**Table 1:** Calculation of the biometric body condition indices.

Index	Abbreviation	Calculation	References
Mass/length ratio	MLR	$M/L$	^9^
Body mass Index	BMI	$M/L^{2} \times {10}^{3}$	^27^
Fulton’s K Index	FKI	$M/L^{3} \times {10}^{6}$	^28,29^
Relative condition	RC	$M/a \times {L}^{b}$, where $a$ and $b$ are obtained from the linear OLS regression $\mathrm{ln}\left(M\right)=\mathrm{ln}\left(a\right)+b\times \mathrm{ln}(L)$	^30^
OLS regression residuals	OLSR	Residuals of the linear OLS regression between $\mathrm{ln}\left(M\right)$ and $\mathrm{ln}(L)$	^31^
Major axis regression residuals	MAR	Residuals of the major axis regression between $\mathrm{ln}\left(M\right)$ and $\mathrm{ln}(L)$	^32^
Reduced major axis regression residuals	RMAR	Residuals of the reduced major axis regression between $\mathrm{ln}\left(M\right)$ and $\mathrm{ln}(L)$	^33^
Scaled mass index	SMI	${M}_{i}\times (L_{0}/L_{i})^{b}$, where $b$ is the slope from a standardized major axis regression between $\mathrm{ln}\left(M\right)$ and $\mathrm{ln}(L)$ and L_0_ is the average of L	^6^

Body mass (M) in grams and total length (L) in mm.

We focused on a selected set of hematology and serum biochemistry biomarkers, as minimally invasive proxies of nutritional physiology (protein and lipid metabolism), physical condition (aerobic capacity and erythrocyte turnover) and health (immune response) (Table 2). To date, most research on the physiological correlates of bBCI has involved destructive approaches such as whole-body macronutrient analysis^6^. The pressing need to integrate animal welfare in wildlife research highlights the need of using the least invasive, non-destructive methods, in line with the 3Rs (replacement, reduction, and refinement) strategy^34^.

**Table 2:** Selected hematology and serum biochemistry parameters and their anticipated relation with body condition.

Parameter (units)	Physiological significance	Expected relation with body condition	References
Albumin (g/dL)	Protein metabolism (main serum protein; decreased in severe malnutrition)	Positive	^35,36^
Urea (mg/dL)	Protein metabolism (protein content of the diet)	Positive	^35–40^
Creatinine (mg/dL)	Protein metabolism (muscular catabolism)	Negative	^35,36,38–40^
Cholesterol (mg/dL)	Lipid metabolism (dietary lipids)	Positive	^35,36,38–40^
Triglycerides (mg/dL)	Lipid metabolism (mobilization of lipid stores)	Negative	^35,36,38–40^
Globulins (g/dL)	Immune system proteins (humoral immune function and acute phase proteins)	Positive	^35,36,39^
Total bilirubin (mg/dL)	Physiological fitness (metabolite of hemoglobin degradation; erythrocyte turnover)	Negative	^35,36,41^
Hemoglobin (g/dL)	Physiological fitness (oxygen carrying capacity of the blood)	Positive	^42,43^

## Results

### Comparison of the biometric body condition indices

**Figure 1:**
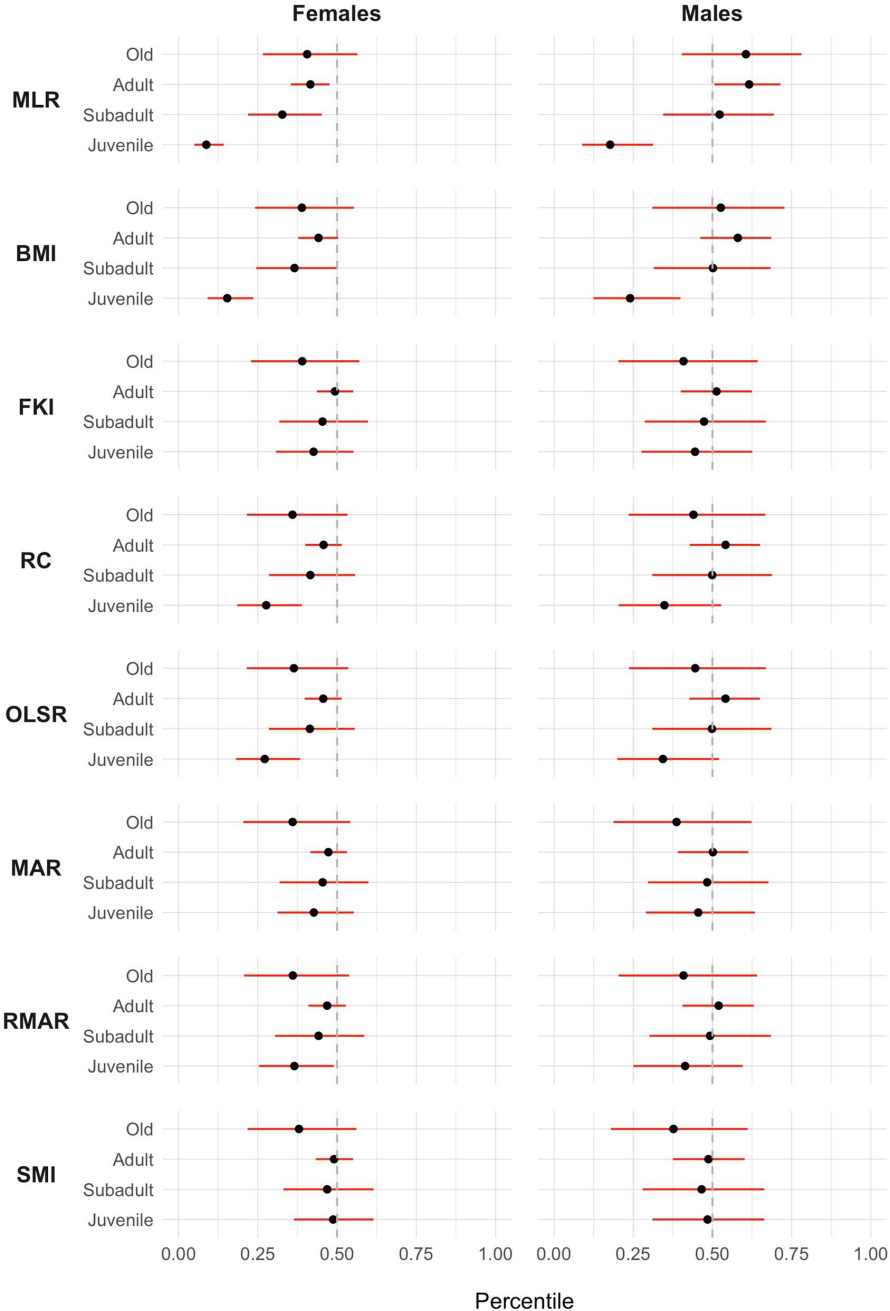
Estimated mean percentile of each body condition index (black dot) and respective 95% credible interval (red line) for each of the sex and age classes, drawn on parameter values from 3000 Markov chain Monte Carlo samples of the posterior distribution. *MLR* Mass/Length Ratio, *BMI* Body Mass Index, *FKI* Fulton’s K Index, *RC* Relative Condition, *OLSR* Ordinary Least Squares Regression Residuals, *MAR* Major Axis Regression Residuals, *RMAR* Reduced Major Axis Regression Residuals, *SMI* Scaled Mass Index.

There was a tendency across all tested bBCI to increase from the young age class, reaching a maximum in adults, and then declining at older life stages (Fig. 1). However, the effect of the age class on body condition varied by bBCI, as age classes were uninformative only for the FKI, MAR and SMI (Table 3).

**Table 3:** Untransformed parameter estimates (mean ± SE) for the first modeling approach, characterizing the relationship between the biometric body condition index (bBCI) percentile and the age classes, sex and both the linear and quadratic terms for the day of the year.

Parameter	MLR	BMI	FKI	RC	OLSR	MAR	RMAR	SMI
Intercept	− **0.34 ± 0.13**	**− **0.23 ± 0.13	**− **0.03 ± 0.12	**− **0.17 ± 0.12	− 0.18 ± 0.12	− 0.11 ± 0.12	− 0.13 ± 0.12	− 0.04 ± 0.12
Age: Juvenile	**− 2.01 ± 0.17**	**− 1.48 ± 0.15**	**− **0.27 ± 0.14	**− 0.79 ± 0.14**	− **0.81 ± 0.14**	− 0.19 ± 0.14	− **0.43 ± 0.14**	− 0.01 ± 0.14
Age: Subadult	**− 0.38 ± 0.14**	**− 0.32 ± 0.16**	**− **0.16 ± 0.18	**− **0.17 ± 0.17	− 0.17 ± 0.17	− 0.07 ± 0.18	− 0.11 ± 0.18	− 0.09 ± 0.18
Age: Old	**− **0.04 ± 0.19	**− **0.22 ± 0.22	**− **0.42 ± 0.26	**− **0.41 ± 0.25	− 0.38 ± 0.24	− 0.47 ± 0.27	− 0.45 ± 0.26	− 0.45 ± 0.27
Sex: Male	**0.81 ± 0.10**	**0.56 ± 0.11**	0.08 ± 0.11	**0.34 ± 0.11**	**0.34 ± 0.11**	0.12 ± 0.11	0.20 ± 0.11	− 0.01 ± 0.12
Day of the year	**− **0.06 ± 0.05	**− 0.13 ± 0.06**	**− 0.17 ± 0.06**	**− 0.15 ± 0.06**	− **0.15 ± 0.06**	− **0.15 ± 0.06**	− **0.16 ± 0.06**	− **0.16 ± 0.06**
(Day of the year)^2^	**0.36 ± 0.05**	**0.31 ± 0.06**	0.11 ± 0.06	**0.22 ± 0.06**	**0.21 ± 0.06**	**0.13 ± 0.06**	**0.16 ± 0.06**	0.10 ± 0.06

Informative parameters are highlighted in bold. Only bBCI with uninformative age and sex estimates were retined for subsequent analyses.Reference classes are females and adults.

*MLR* Mass/Length Ratio, *BMI* Body Mass Index, *FKI* Fulton’s K Index, *RC* Relative Condition, *OLSR* Ordinary Least Squares Regression Residuals, *MAR* Major Axis Regression Residuals, *RMAR* Reduced Major Axis Regression Residuals, *SMI* Scaled Mass Index.

Likewise, sex was informative for MLR, BMI, RC and OLSR, with higher body condition values for males than for females (Table 3, Fig. 1). Consequently, our modeling procedure shows that the Fulton’s K Index (FKI), Major Axis Regression Residuals (MAR) and Scaled Mass Index (SMI) were the only bBCI effectively controlling for the effect of sex and age, hence these were retained for the subsequent analyses.

**Figure 2:**
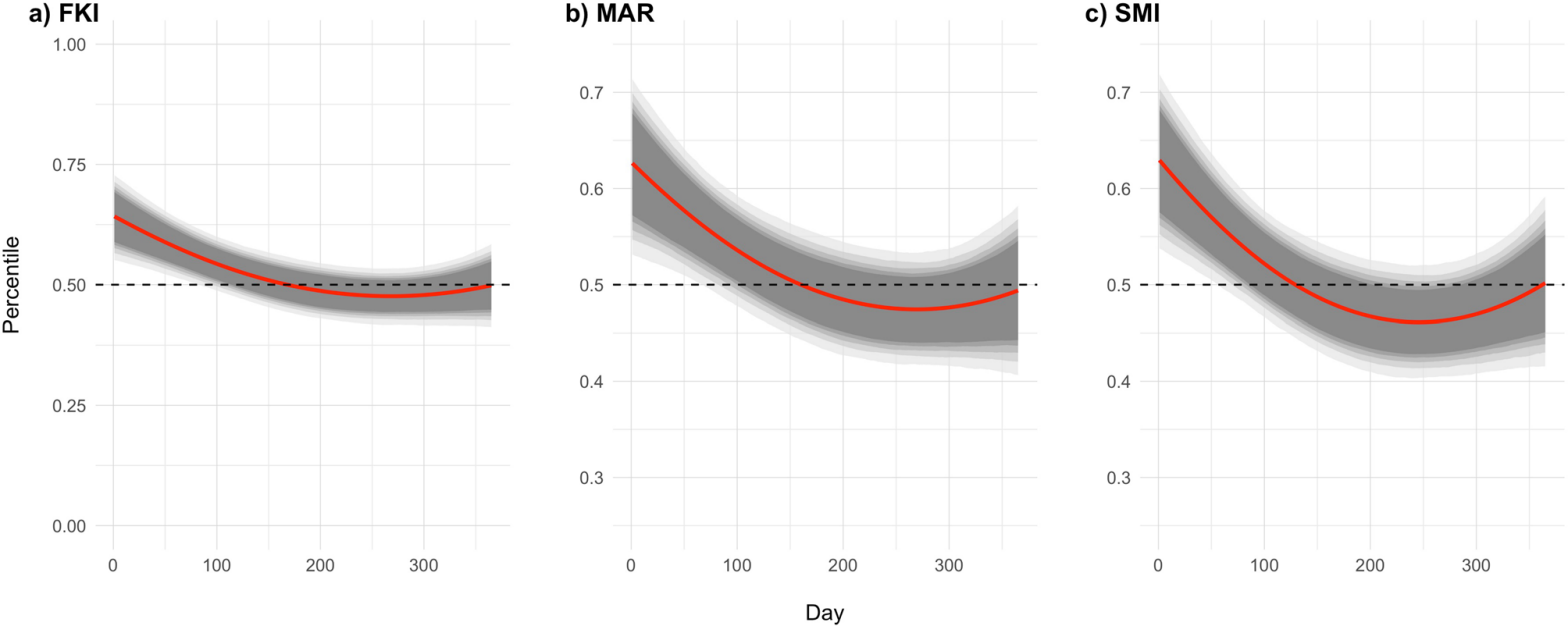
Selected body condition indices according to the day of the year. Fultons’s K Index (**A**), Major Axis Regression Residuals (**B**) and Scaled Mass Index (**C**). Mean predicted relationship curve (red line) and respective 75%, 80%, 85%, 90% and 95% credible intervals (different shades of grey areas) drawn on parameter values from 3,000 MCMC samples of the posterior distribution.

The selected bBCI show a similar pattern of variation in body condition with the day of the year (Table 3). The model results support that, despite some variability, Iberian carnivores tend to exhibit higher body condition values early in the year and then body condition decreases, reaching its lowest in late summer and increasing towards the end of the year (Fig. 2).

Further details about the descriptive statistics of each species-specific bBCI and their fitted distributions can be found in the Supplementary Information (Table S5 and Fig. S3).

### Effect of the hematology and serum biochemistry parameters

**Figure 3:**
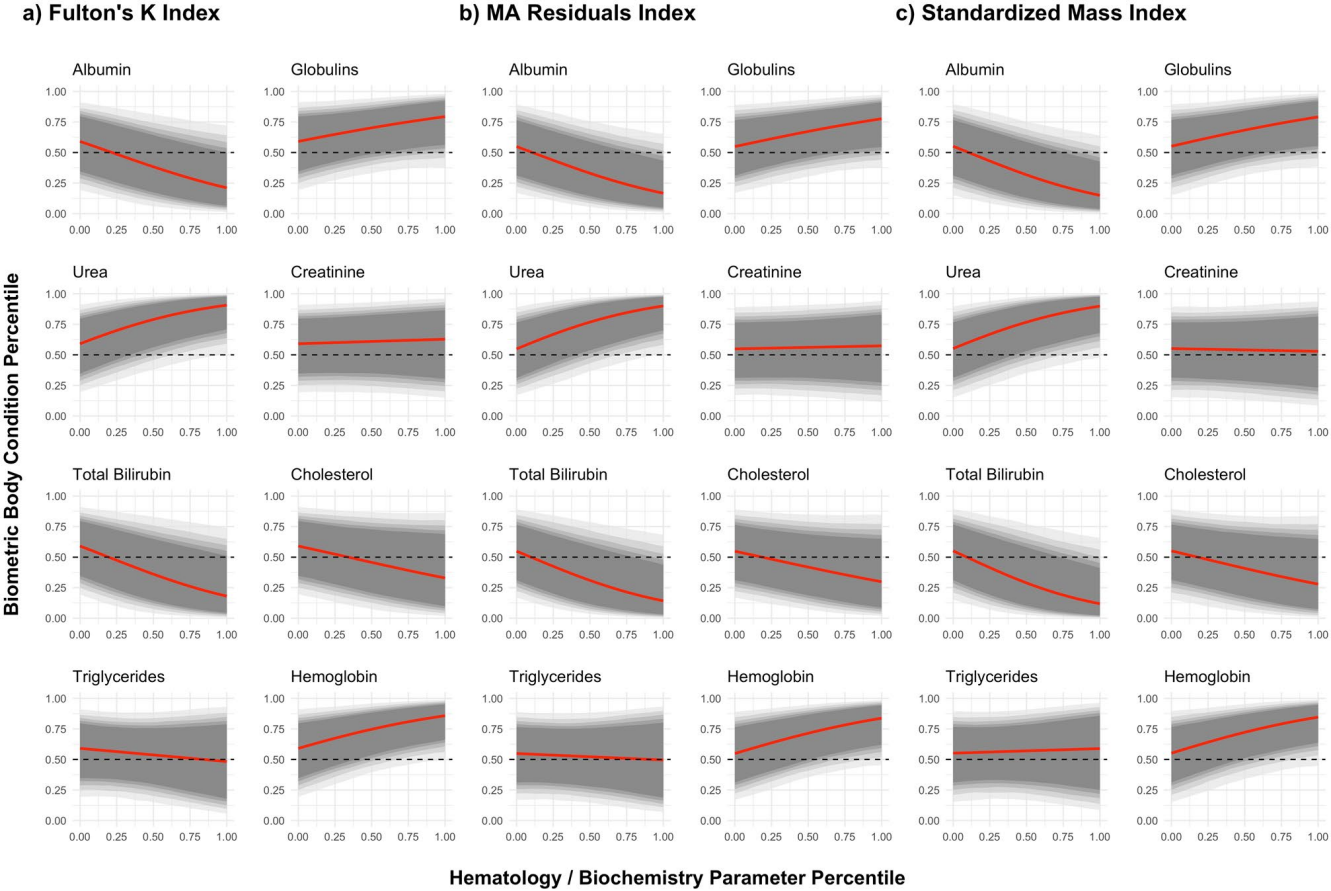
Relationship between the selected biometric body condition indices according to the observed variation in hematology and biochemistry parameters. Fulton’s K Index (**A**), Major Axis Regression Residuals (**B**) and Scaled Mass Index (**C**). Mean predicted relationship curve (red line) and respective 75%, 80%, 85%, 90% and 95% credible intervals (different shades of grey areas) drawn on parameter values from 3000 MCMC samples of the posterior distribution.

The models support that the most informative biochemistry parameters in explaining the variation in bBCI are albumin, urea and total bilirubin (Table 4, Fig. 3). Particularly, albumin and total bilirubin had the strongest negative effect size (Albumin: ${ES}_{SMI} = -1.66$, ${ES}_{MAR} = -1.76$, ${ES}_{FKI} = -1.67$; Total bilirubin: ${ES}_{SMI} = -1.70$, ${ES}_{MAR} = -1.79$, ${ES}_{FKI} = -1.62$), while urea revealed a strong positive effect on the bBCI percentile (Urea: ${ES}_{SMI} = 1.61$, ${ES}_{MAR} = 1.85$, ${ES}_{FKI} = 1.71$) (Fig. 3). Hemoglobin, globulins and cholesterol were moderately informative biochemical parameters of the overall carnivores’ bBCI, revealing effect size values ranging between 0.9 and 1.5. Of these, hemoglobin concentration and globulins tended to increase with increasing bBCI values, whereas cholesterol tended to decrease (Table 4, Fig. 3). The remaining biochemistry parameters (triglycerides and creatinine) were largely uninformative as they presented weak associations with all the bBCI ($|ES|< 0.5$) (Fig. 3).

**Table 4:** Untransformed parameter estimates (mean ± SE) for the first modeling approach, characterizing the relationship between each physiological parameter and the retained body condition indices.

Parameter	FKI	MAR	SMI
Intercept	0.37 ± 0.94	0.20 ± 0.92	0.21 ± 0.96
Albumin	− **1.69 ± 1.01**	− **1.80 ± 1.02**	− **1.94 ± 1.17**
Urea	**1.91 ± 1.12**	**2.00 ± 1.08**	**1.98 ± 1.23**
Creatinine	0.16 ± 0.86	0.10 ± 0.81	− 0.09 ± 0.92
Cholesterol	− 1.08 ± 1.13	− 1.05 ± 1.10	− 1.16 ± 1.21
Triglycerides	-0.44 ± 1.35	-0.21 ± 1.31	0.15 ± 1.41
Globulins	0.98 ± 1.05	1.05 ± 0.99	1.13 ± 1.15
Total bilirubin	− **1.88 ± 1.16**	− **1.99 ± 1.11**	− **2.21 ± 1.30**
Hemoglobin	1.43 ± 1.03	1.45 ± 1.01	1.50 ± 1.13

Informative parameters are highlighted in bold.

*FKI* Fulton’s K Index, *MAR* Major Axis Residuals, *SMI* Scaled Mass Index.

## Discussion

Using non-destructive methods, we assess the hematology and serum chemistry correlates of bBCI in Southern European mammalian carnivores and provide evidence that these biometric indices are a composite measure of several physiologic processes. Our results support that bBCI should be considered as a useful surrogate of individual’s overall nutritional physiology and physical fitness.

Biometric BCI should account for the growth process, allowing to compare individuals with different body sizes, sex or developmental stages^1,6,12^. However, out of the eight commonly used bBCI analyzed, only Fulton’s K Index^28^, Major Axis Regression Residuals^32^ and the Scaled Mass Index^6^ fulfill these requirements.

Fulton’s K Index is the only ratio-based index among the selected bBCI. Ratio-based bBCI are of widespread use^2^, despite being widely criticized^12^. At the core of the criticisms to ratio-based condition indices is the strong correlation with animals’ size based on the underlying relationship between mass and body size^11^. The body mass of an animal equals its density multiplied by its volume, therefore, if density does not change, an animal’s volume is expected to increase in proportion to length cubed^11^. The present study supports that by making the exponent of length equal to the length-to-mass scaling component (i.e. 3), FKI is relatively insensitive to the sex and age class of carnivores, hence it could provide a useful tool for monitoring body condition in these species.

Indices of body condition based on the residuals of a linear regression between mass and body size are uncorrelated with the later^11^ and thus are theoretically more appropriate as biometric body conditions indices^6^. Interestingly, we found that only the Major Axis Regression Residuals index exhibited the targeted requirement of independence from the sex and age class. The major axis regression assumes the existence of measurement error in both mass and length variables and its main purpose is to estimate the slope of the linear regression between the two variables^44^. Conversely, the SMI is based on the slope of mass against length, estimated by a reduced major axis regression^6^. It has been shown to have suitable properties for a bBCI, such as providing a precise estimate of the slope of the relation between mass and length while accounting for different magnitudes of measurement error and variability^6,12,44^.

All analyzed bBCI showed a tendency for an increase from the juveniles to subadults to adults, although not informative in the selected indices. This tendency could result from these bBCI imperfectly controlling for the growth process or to potential changes in body condition among age classes, due to differences in metabolic rates or body composition. Interestingly, there is also a general tendency for a decrease in the bBCI in older animals, which could reflect a decline in the capacity of the organism to maintain optimal functionality of its vital systems^4^. Such pattern can be due to senescence^45^, eventually mediated by a terminal investment life-history strategy^46^, whereby older individuals invest high energy levels into increasing late life-stage reproductive success^7^. The potential of bBCI as biomarkers of senescence in wildlife deserves further study.

While controlling for growth, sex and age, bBCI should be sensitive to changes in the true body condition of an organism. We argue that seasonal fluctuations in resource availability translate into annual cycles of body condition and thus provide a suitable framework to assess the sensitivity of bBCI to these changes. Seasonal fluctuations in body condition were previously described in free-ranging ungulates^14^ and could also occur in carnivores^20,21^. In the temperate Mediterranean bioregions of the Iberian Peninsula, where 88% of our sample originates from, these seasonal cycles translate into maximum net primary productivity in the spring and autumn, and minimum in the summer^47,48^. The general seasonal dynamics of the three selected bBCI broadly match these expectations, showing a nadir in late summer^13^ and increasing throughout the final stages of the year (Fig. 2). The decreased body condition during spring was expected, as the most energy-intensive stages of the reproductive cycle, at least for females (late gestation and lactation) take place during this period^49^ and offset the increased net primary productivity in this season.

Biometric BCI are often considered second-order surrogates of Darwinian fitness, being used as an estimate of body lipid content, which is assumed to be related to fitness^2^. However, the link between bBCI and lipid content has been shown to be a fragile one^1,8,15,49^. Our results revealed that the most informative hematology and serum biochemistry parameters explaining the observed variability in bBCI were proxies of protein metabolism (albumin and urea) and physical fitness sensu Caspersen et al.^22^ (total bilirubin). Other parameters, proxies of lipid metabolism (cholesterol), immune function (globulins) and physical fitness (hemoglobin concentration) only moderately contributed to explaining variability in the bBCI.

Overall, surrogates of physical fitness (total bilirubin and hemoglobin concentration) fit the initial predictions in their observed relationship with the selected bBCI. The models support that Southern European carnivores in the higher percentiles of the bBCI species-specific distribution tend to have higher hemoglobin concentration. This pattern was also found elsewhere in black bears *Ursus americanus*^50^. Higher hemoglobin levels provide increased aerobic capacity^49^, hence presumably enhancing the individual’s aptitude to carry out physically demanding tasks such as predation of elusive prey^3^. Conversely, total bilirubin, which is a by-product of hemoglobin catabolism^51^, was predicted to decrease with increasing body condition due to a diminished erythrocyte turnover^41^. Furthermore, mild albumin-bound bilirubinemia has a strong antioxidant effect^52^, which could counteract the increased free-radical production in organisms on a negative energy balance^53^. The present results corroborate these predictions, suggesting that individuals with higher bBCI values were more physically fit.

Globulins was the only physiological parameter related to immune function, (and comprise mostly acute phase proteins and immunoglobulins^54^, both involved in the physiological response to infection and inflammation^35^. Mounting a proper immune response requires allocation of significant amounts of energy, which trade-off with other traits such as reproduction and survival^24^. Hence, it is not surprising to find a tendency for higher globulin concentration in animals with higher body condition, although this parameter was not informative^26,55^.

Two parameters related to protein metabolism (albumin and urea) were also strongly correlated with the bBCI. The covariation of urea with the selected bBCI fits the initial predictions, suggesting that carnivores with higher body condition tend to have a protein-rich diet^21,37,56^. Animals may employ diverse preingestive mechanisms (e.g. prey selection) for regulating their body composition^20^. Hence, body condition might be regulated by specific components of their diets rather than overall caloric content of their food^7,21^. Surprisingly, albumin showed a negative relation with bBCI, possibly because the postulated positive relation of albumin concentration only applies to extreme values of body condition, such as decreased albumin concentration in starving animals^36^. The main physiological function of albumin is to set the osmotic pressure of the plasma, transporting metabolites, antioxidant defense, and in supplying amino acids to tissues when on a negative protein balance^35^. Oxidant production is influenced by diet and increases when on a negative energy balance^53^, so higher albumin concentration would be advantageous for animals in lower body condition or on a protein-deficient diet.

Cholesterol was not found to be positively correlated to body condition, as cholesterol concentration tended to decrease from lower to higher values of bBCI in Southern European carnivores. Cholesterol is essential as a cellular membrane component and a precursor to bile acids^35^, and has been shown to be present in higher concentrations in the serum of cats and other mammals on a lipid-rich diet, and in lower levels when on a protein-rich diet^56,57^. Studies are needed on the relation between bBCI and the high and low-density lipoproteins, components of the total serum cholesterol concentration.

Summarizing, the overall nutritional physiology metabolic patterns suggest a gradient from protein-poor diets (high Cholesterol and low Urea) to protein-rich diets (high Urea and low Cholesterol) with increasing bBCI^13,21,56,57^. This is accompanied by a trend towards increasing physical fitness (higher aerobic capacity and lower erythrocyte turnover) and immune function^3,26^. These characteristics are likely advantageous for carnivores that depend on active predation for foraging^3^.

The strength of our conclusions is tempered by the relatively small number of carnivores, dispersed over 6 species, for which a complete hematology and serum biochemistry dataset was available. Furthermore, there is a surprising absence of published reference intervals for some physiological parameters of abundant and widespread carnivore species (Table S4), which might undermine the data standardization. While we were limited to testing a priori hypothesis, metabolomic approaches might allow a broader view of the physiological significance of bBCI.

This study provides evidence supporting the initial hypothesis that bBCI are a composite measure of several physiological processes, namely nutritional physiology (particularly protein metabolism) and physical fitness in mammalian carnivores. The complexity of factors underlying the bBCI should not be viewed as hindering their usefulness, but rather as one more strength to their use, allowing to integrate in one easily obtained metric, many aspects of physiology relevant for ecology and wildlife conservation^10^. Furthermore, this study shows that many of the commonly used bBCI do not control for the effect of the age and sex in Southern European carnivores. We suggest that the seasonal pulses in resource availability can be used to assess the sensitivity of bBCI to variations in the true body condition of carnivores. We recommend that future in-depth studies are needed on the likely complex relation between body condition and nutritional physiology, particularly using metabolomic approaches^56^. Additionally, the relation between body condition and components of Darwinian fitness, such as survival and reproduction, need to be assessed by longitudinal studies in Southern European mammalian carnivores.

## Methods

### Animal capture and handling

Free-ranging carnivores (n = 434) from six species were captured across 25 study areas in the Iberian Peninsula (Fig. S1), mainly in the Mediterranean bioregion^47,48^: 104 red foxes, 100 Egyptian mongooses, 82 common genets, 72 stone martens, 53 Iberian wolves and 23 European wildcats. The dataset includes biometric data obtained from 34 fresh cadavers of wolves killed by sudden causes of death (run-over by vehicle or shot), provided by the Portuguese dead wolf monitoring system (*Sistema de Monitorização de Lobos Mortos/ICNF*). Four of the European wildcats were identified by genetic analysis as F1 wildcat x domestic cat hybrids^58^. However, they were retained in the dataset as the domestic and wildcats are conspecifics, and because these were presumed free-ranging animals.

Summary statistics regarding sex, age and seasonal distribution of the data are presented as Supplementary Information (Table S1 and Fig. S2). Briefly, the sample was evenly distributed between sexes (217 females and 216 males), but biased towards adults (256 adults vs 57 subadults, 97 juveniles and 24 old animals).

Captures were performed using three different methods: (i) box-trapping, using double- and single-door box-traps (Tomahawk 109, USA, and VK1150310, Portugal); (ii) neck-snaring with stop (Collarum, Wildlife Control Supplies, EUA); and (iii) leg-hold snaring (Belisle, Edouard Belisle, Canada). Food or scent lures were used as attractants. Traps were checked once or twice daily and leg-hold snares were operated coupled with remote satellite trap-alarms (TT2 Globastar Trap Transmitter, Vectronic Aerospace, Germany). Whenever weather conditions were predicted to be extreme (maximum daily temperature > 35ºC or total daily precipitation > 10 mm), box-traps and neck snares were deactivated to minimize exposure of the captured carnivores.

All trapping procedures were licensed by the Portuguese nature conservation authority *Instituto de Conservação da Natureza e Florestas*, Portugal (Licenses nr. 395/2011/CAPT/MANUS e 362/2012/CAPT/MANUS, 338/2007/CAPT, 258/2008/CAPT, 286/2008/CAPT, 260/2009/CAPT, 332/2010/MANU, 333/2010/CAPT, 336/2010/MANU, 26/2012/MANU and 72/2014/CAPT) and the Castilla-La Mancha Regional Government, Spain (Licenses nr. 02-227/RN-52, PREG-05-23 and PR-2013-05-04), according to Portuguese (*Decreto-Lei* 113/2013), Spanish (*Real Decreto* 53/2013) and European legislation (Directive 2010/63/EU), and followed international standards on the use of wild animals for scientific research^59,60^.

The protocol for the manipulation of trapped wolves was previously described in Santos et al.^61^. Mesocarnivores captured with box-traps were transferred to a restraint-cage, equipped with a sliding wall and covered by a dark blanket to reduce capture stress. All trapped carnivores were chemically immobilized by the intramuscular injection of species-adapted doses of ketamine (Imalgene, Merial, France) and medetomidine (Domitor, Merial, France). Immobilization was reversed by the intramuscular injection of atipamezole (Revertor, Merial, France).

Blood samples were collected by venipuncture of the cephalic or saphenous veins 4–54 min after administration of anesthesia and preserved in EDTA and clotting tubes, kept refrigerated and protected from sunlight and excessive agitation. The complete set of hematology and serum biochemistry parameters was available for 42 animals (19 Egyptian mongooses, 10 red foxes, 6 wolves, 4 common genets, 2 wildcats and 1 stone marten). The age of each animal was estimated by dental eruption and wear, and classified as juveniles (deciduous teeth present), subadults (only permanent teeth, no wear detectable), adults (slight to moderate wearing of the teeth) and old (heavily worn teeth)^62–64^. Gender was assessed by inspection of genitalia.

### Body condition indices

We obtained three body structural measures with a metric tape (1 mm precision): (i) total length, from snout to the distal end of the last tail vertebrae; (ii) body length, from snout to the proximal end of the first tail vertebrae; (iii) tarsus length, from the proximal tarsus to the distal end of the longest digit (not including claw). The body mass of the captured animals was measured with 10 g precision with a scale (HCB20K10, Kern & Sohn, Germany). Following Peig & Green^6^, total length was selected as the body size measurement used to estimate the bBCI, due to the stronger correlation with body mass (Table S2).

We then estimated eight commonly used bBCI: Mass/Length Ratio (MLR), Body Mass Index (BMI), Fulton’s K Index (FKI), Relative Condition (RC), Ordinary Least Squares Regression Residuals (OLSR), Major Axis Regression Residuals (MAR), Reduced Major Axis Regression Residuals (RMAR), and Scaled Mass Index (SMI) (Table 1).

### Hematology and serum chemistry

The following set of hematology and serum chemistry parameters was selected based on their physiological significance and relative insensitivity to sampling effects such as capture-related stress^65^: albumin, urea, creatinine, cholesterol, triglycerides, globulins, total bilirubin and hemoglobin (Table 2).

Hemoglobin concentration was determined by the alkaline hematin method^66^, and the absorbance at 580 nm determined in spectrophotometer (Ultrospec 3100 Pro, Amersham Biosciences, UK). Briefly, 20µL of blood in EDTA was diluted 1:200 in NaHO 0.1 M with 2.5% Triton X to lyse the erythrocytes and convert hemoglobin to alkaline hematin. Calibration curves were calculated by applying the same protocol for alkaline hematin to samples from captive red foxes (n = 8), Egyptian mongooses (n = 6), genets (n = 2), and pet dogs (n = 8) and cats (n = 6), whose hemoglobin concentration was simultaneously determined by a hematology analyzer (Sysmex XT-2000iV, Sysmex Corporation, Japan) in a commercial laboratory (Inno, Portugal). Hemoglobin concentration and absorbance were log-transformed, and their ordinary least squares linear relation was estimated and used to convert absorbance readings to hemoglobin concentration (R^2^ = 0.922).

Blood in clotting tubes was centrifuged at 1430×*g* for 10 min (CNT800D, Quirumed, Spain), on the day after capture, and serum obtained and kept at -20 °C until analysis. Serum was analyzed for chemistry parameters in a commercial laboratory (Inno, Portugal) using a Mindray BS380 (Mindray Medical International, China) clinical biochemistry analyzer. Briefly, proteins (Alb and Glob) were quantified by electrophoresis and metabolites’ (Urea, Crea, TBil, Chol and Trig) concentrations were determined by several methods as available from the manufacturer (www.mindray.com/).

### Data standardization

In order to be able to pool data from different species, all the observations were standardized to their species-specific percentile. This approach assumes that individuals of different species in the same percentile of their species-specific bBCI distribution have equivalent body condition.

The species-specific distribution of each bBCI was estimated by randomly generating 10,000 simulated values from a distribution fitted to the observations (Table S3). The candidate distributions were obtained by maximum goodness-of-fit, using the R^67^ package “fitdistriplus”^68^ and selected by their log-likelihood and Anderson–Darling test^69^ (Table S3), and through a visual assessment of their empirical and theoretical probability densities functions, P–P and Q–Q plots. The comparison between the distributions of each observed sample and the randomly generated values was performed with the Kolmogorov–Smirnov test^70^ (all p > 0.357—Table S3). From the derived distributions we estimated the percentile of each captured animal in the distribution range of each bBCI.

The species-specific distribution of each hematology and serum chemistry parameter was estimated by randomly generating 1000 simulated values from a posterior distribution obtained by combining in a probability tree, the published reference ranges and the observations obtained in the present study (Table S4), using the R package “mc2d”^71^. Reference ranges were modeled as expert opinion (beta-PERT distribution) from their mean, minimum and maximum values^72^. The relative weight of the reference range and of our observations in the posterior distribution was determined by the respective sample sizes. The comparison between the distributions of each observed sample and the posterior distribution was performed with the Kolmogorov–Smirnov test^70^. From the derived distributions we estimated the percentile of each captured animal in the distribution range of each hematology and serum biochemistry parameter.

### Statistical modeling

Spearman’s rank correlation was used to test for collinearity ($\rho \ge 0.7$) among all covariates. When correlated covariates were detected, the one with the greatest correlation with the response variable was retained^70^. The relative importance of the model parameters was assessed by analysing their posterior distributions. Parameters were considered informative if the 95% credible interval of the posterior distribution did not include zero, and their respective effect sizes (ES) were calculated by weighting the posterior mean over its precision, such that $ES={\widehat{\beta} }/SE$.

The premise that body condition should control for the sex and age class^12^ was tested using a Bayesian Generalized Linear Modeling (GLM) approach, with an additional error term to account for inter-species variability. Each level of the age-specific and sex-specific covariates was coded as a binary dummy variable whereby ‘1’ denotes an individual belonging to that specific class and ‘0’ denotes the opposite. The classes considered as reference were ‘adults’ for age, and ‘females’ for sex. Therefore, the estimates for these classes were included in the models’ intercept, and the parameter estimates for the remaining classes were calculated as relative differences to the reference classes. The other ages classes included in the models were ‘juveniles’, ‘sub-adults’ and ‘old’ animals.

The seasonal variation in bBCI was modeled by including a z-score standardized form of the linear and quadratic terms for the day of the year, therefore allowing for local maxima/minima across the year cycle. Given that the response variable (i.e. bBCI percentile) corresponds to non-binomial data bounded between 0 and 1, we formulated the model in the logit scale^73^, such that:

$$logit\left(pBCI\right)={\beta 0}_{i}+ {\beta 1}_{i}* {pCov1}_{i}+ {\beta 2}_{i}* {pCov2}_{i}+\dots + {\varepsilon }_{species},$$

where $pBCI$, $pCov1$ and $pCov2$ denote the percentiles of the bBCI, and of the respective first and second covariates, and ${\varepsilon }_{species}$ denotes the error term accommodating inter-species variability. The bBCI effectively controlling for the effect of age and sex classes^12^, i.e. those whose 95% credible interval of the posterior distribution of the parameter estimates did not overlap zero, were retained for subsequent analyses.

The percentile of each carnivore’s bBCI in the species-specific bBCI distribution was modeled as a function of the above described hematology and serum biochemistry parameters, also recorded as percentile in their respective species-specific distributions.

To test the hypothesis that variation in blood hematology and biochemistry correlates with carnivores’ body condition we fitted a Bayesian Generalized Linear Model (GLM), with an error for inter-species variability. The dependent and independent variables were the species-specific percentiles of bBCI and hematology and biochemistry parameters, formulated on the logit scale.

Posterior distributions were obtained using Markov chain Monte Carlo (MCMC) implemented in JAGS (version 4.3.0), using “R2Jags”^74^ through “rjags”^75^ in R software^67^. We generated three MCMC with 30,000 iterations after 10,000 iterations burn-in and thinned by 10. Prior distributions for each of our model parameters consisted of a near-flat normal distribution of mean 0 and SD of 1000 on the logit scale. We assessed model convergence from a visual inspection of chain trace plots and from the Gelman-Rubin statistic, where R-hat < 1.1 suggested convergence^76^.

## Supplementary information

Supplementary Information.

## Data Availability

The datasets generated during and/or analysed during the current study are available from the corresponding authors on reasonable request.
