# Supplementary material for: Protein metabolism and physical fitness are physiological determinants of body condition in Southern European carnivores
Source: Sci Rep. 2020 Sep 25;10:15755. doi: 10.1038/s41598-020-72761-6 (PMC7519690; doi:10.1038/s41598-020-72761-6)
Supplement: Supplementary file 1 — Supplementary Information. [file 41598_2020_72761_MOESM1_ESM.docx]

**Protein metabolism and physical fitness are physiological determinants of body condition in Southern European carnivores**

Nuno Santos ^1^, Mónia Nakamura ^1,2^, Helena Rio-Maior ^1^, Francisco Álvares ^1^, Jose Ángel Barasona ^3^, Luís Miguel Rosalino ^4^, Maria João Santos ^5^, Margarida Santos-Reis ^4^, Pablo Ferreras ^6^, Francisco Díaz-Ruíz ^6,7^, Pedro Monterroso ^1^

**Supplementary Information**

| **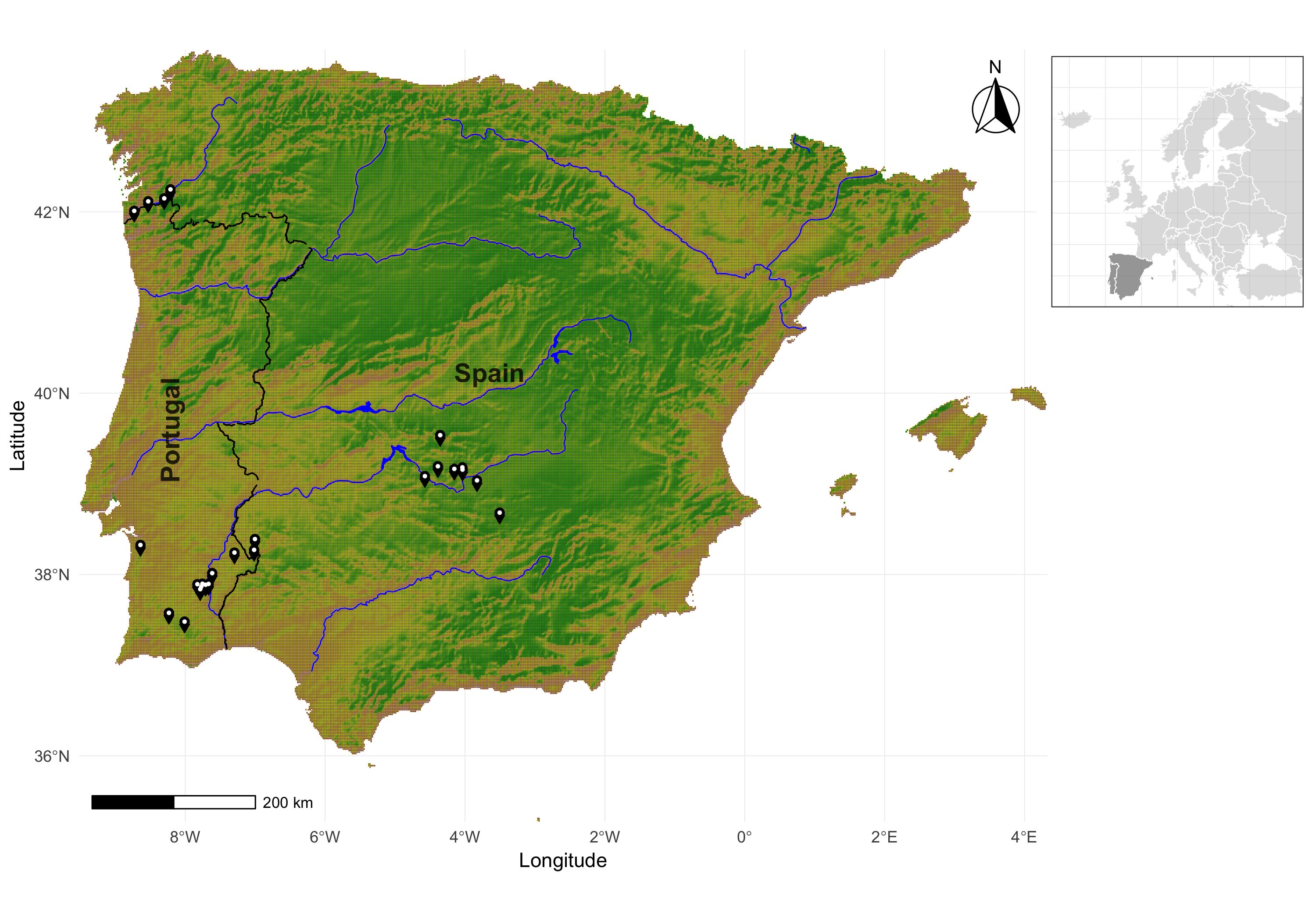** |
| --- |
| **Figure S1.** Location of the study areas in the Iberian Peninsula. Map created using QGIS 2.18.0 (http://qgis.org). |

| **Species** | **Sex** | | | **Age** | | | |
| --- | --- | --- | --- | --- | --- | --- | --- |
|  | **Female** | **Male** | **Juvenile** | | **Subadult** | **Adult** | **Old** |
| Egyptian mongoose (*Herpestes ichneumon*) | 55 | 44 | 23 | | 8 | 61 | 8 |
| Red fox (*Vulpes vulpes*) | 54 | 50 | 24 | | 28 | 51 | 1 |
| Common genet (*Genetta genetta*) | 36 | 46 | 21 | | 10 | 43 | 8 |
| Stone marten (*Martes foina*) | 27 | 45 | 9 | | 1 | 55 | 7 |
| Iberian wolf (*Canis lupus*) | 33 | 20 | 18 | | 5 | 30 | 0 |
| European wildcat (*Felis silvestris*) | 12 | 11 | 2 | | 5 | 16 | 0 |
| **Total** | 217 | 216 | 97 | | 57 | 256 | 24 |

**Table S1.** Summary statistics of the Iberian carnivore sample according to age and sex.

| 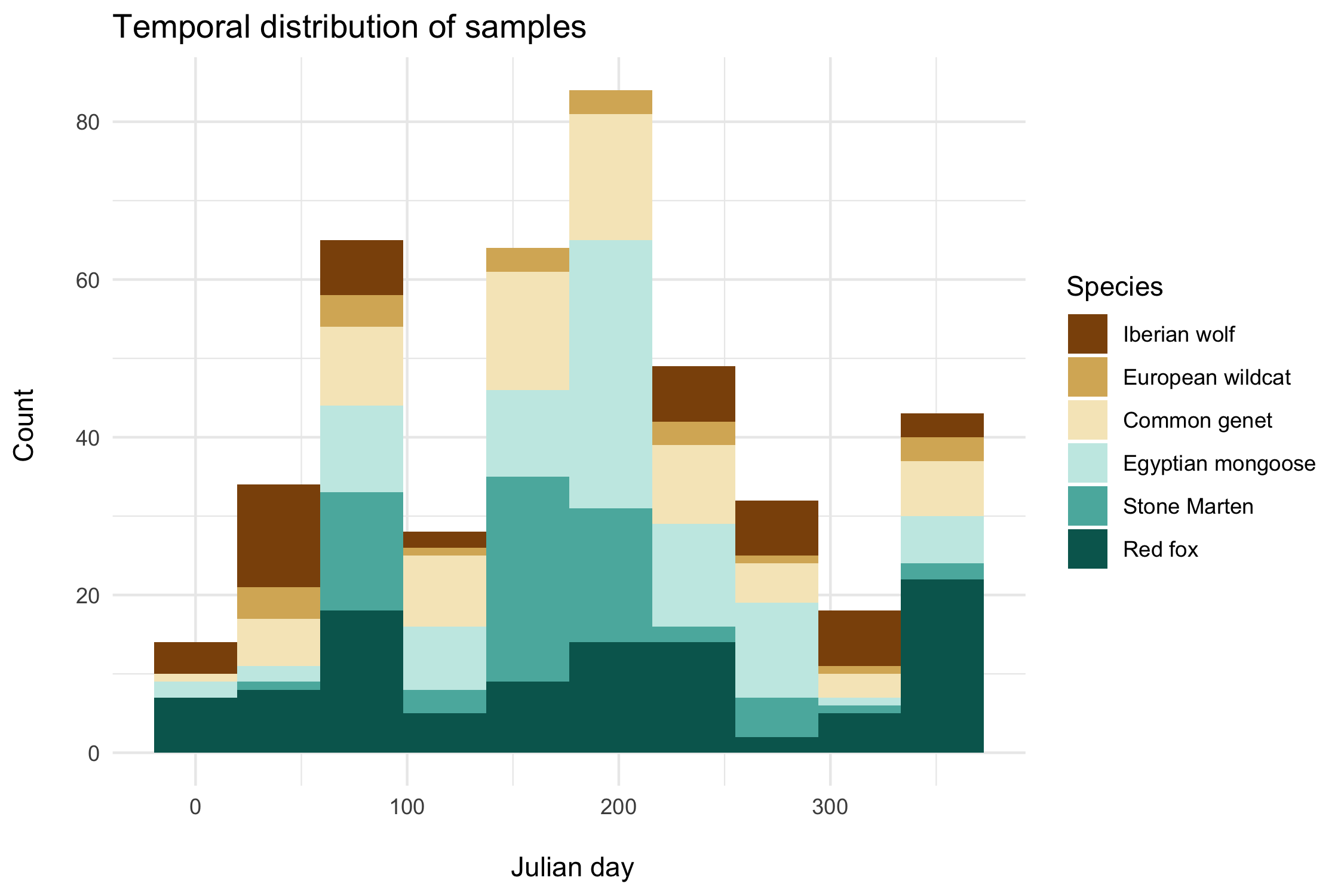 |
| --- |
| **Day of the year** |

**Figure S2.** Distribution of the sample across the year. The day of the year of the trapping or death was assigned to each carnivore.

| **Species** | **Total length** | **Body length** | **Tarsus length** |
| --- | --- | --- | --- |
| Red fox *Vulpes vulpes* | **0.920** | 0.884 | 0.658 |
| Egyptian mongoose *Herpestes ichneumon* | **0.907** | 0.835 | 0.818 |
| Common genet *Genetta genetta* | **0.959** | 0.909 | 0.722 |
| Stone marten *Martes foina* | **0.826** | 0.742 | 0.806 |
| European wildcat *Felis silvestris* | **0.869** | 0.855 | 0.640 |
| Iberian wolf *Canis lupus* | **0.889** | 0.676 | 0.635 |

**Table S2.** Pearson’s correlation coefficient between body mass and body size measurements. Body mass and size were log transformed. In bold the strongest correlation for each species.

| **Species** | **bBCI** | **Distribution family** | **Parameters** | **Anderson-Darling statistic** | **Kolmogorov-Smirnov**  **simulated data (p)** |
| --- | --- | --- | --- | --- | --- |
| *Herpestes ichneumon* | MLR | Weibull | Shape=4.630, Scale=2.350 | 0.358 | 0.642 |
|  | BMI | Gamma | Shape=27.663, Rate=11.247 | 0.143 | 0.997 |
|  | FKI | Logistic | Location=2.816, Scale=0.256 | 0.559 | 0.877 |
|  | RC | Logistic | Location=0.997, Scale=0.0954 | 0.616 | 0.755 |
|  | OLSR | Normal | Mean=-0.007, Sd=0.159 | 0.304 | 0.883 |
|  | MAR | Normal | Mean=-0.004, Sd=0.155 | 0.705 | 0.593 |
|  | RMAR | Normal | Mean=-0.005, Sd=0.151 | 0.426 | 0.971 |
|  | SMI | Logistic | Location=7.472, Scale=0.093 | 0.570 | 0.597 |
| *Vulpes vulpes* | MLR | Weibull | Shape=4.976, Scale=5.254 | 0.531 | 0.867 |
|  | BMI | Logistic | Location=4.850, Scale=0.475 | 0.554 | 0.712 |
|  | FKI | Logistic | Location=4.913, Scale=0.462 | 1.885 | 0.857 |
|  | RC | Logistic | Location=1.003, Scale=0.092 | 0.574 | 0.918 |
|  | OLSR | Normal | Mean=-0.002, Sd=0.157 | 0.568 | 0.935 |
|  | MAR | Logistic | Location=-0.009, Scale=0.090 | 0.292 | 0.953 |
|  | RMAR | Logistic | Location=-0.005, Scale=0.092 | 0.272 | 0.985 |
|  | SMI | Logistic | Location=8.389, Scale=0.092 | 0.857 | 0.934 |
| *Genetta genetta* | MLR | Weibull | Shape=5.886, Scale=1.700 | 1.509 | 0.376 |
|  | BMI | Weibull | Shape=7.318, Scale=1.891 | 0.313 | 0.890 |
|  | FKI | Logistic | Location=2.037, Scale=0.142 | 0.655 | 0.767 |
|  | RC | Lognormal | Meanlog=-0.009, Sdlog=0.111 | 0.564 | 0.813 |
|  | OLSR | Normal | Mean=-0.009, Sd=0.111 | 0.564 | 0.767 |
|  | MAR | Normal | Mean=-0.009, Sd=0.109 | 0.591 | 0.986 |
|  | RMAR | Normal | Mean=-0.009, Sd=0.108 | 0.568 | 0.959 |
|  | SMI | Logistic | Location=7.116, Scale=0.066 | 0.409 | 0.991 |
| *Martes foina* | MLR | Weibull | Shape=6.755, Scale=1.972 | 0.289 | 0.933 |
|  | BMI | Weibull | Shape=8.372, Scale=2.853 | 0.366 | 0.994 |
|  | FKI | Gamma | Shape=47.347, Rate=11.779 | 0.126 | 0.999 |
|  | RC | Normal | Mean=1.009, Sd=0.137 | 0.197 | 0.993 |
|  | OLSR | Normal | Mean=0.005, Sd=0.137 | 0.296 | 0.964 |
|  | MAR | Normal | Mean=0.001, Sd=0.154 | 0.150 | 0.999 |
|  | RMAR | Normal | Mean=0.004, Sd=0.141 | 0.167 | 0.955 |
|  | SMI | Gamma | Shape=2351.817, Rate=330.446 | 0.153 | 0.992 |
| *Canis lupus* | MLR | Weibull | Shape=5.357, Scale=21.021 | 0.517 | 0.693 |
|  | BMI | Weibull | Shape=7.271, Scale=13.677 | 0.252 | 0.909 |
|  | FKI | Logistic | Location=8.740, Scale=0.815 | 0.317 | 0.922 |
|  | RC | Weibull | Shape=8.167, Scale=1.072 | 0.369 | 0.626 |
|  | OLSR | Logistic | Location=0.018, Scale=0.088 | 0.769 | 0.583 |
|  | MAR | Logistic | Location=0.003, Scale=0.099 | 0.333 | 0.768 |
|  | RMAR | Logistic | Location=0.011, Scale=0.093 | 0.472 | 0.924 |
|  | SMI | Logistic | Location=10.189, Scale=0.116 | 0.398 | 0.995 |
| *Felis silvestris* | MLR | Normal | Mean=4.131, Sd=1.028 | 0.173 | 0.999 |
|  | BMI | Weibull | Shape=7.960, Scale=5.294 | 0.472 | 0.955 |
|  | FKI | Logistic | Location=6.001, Scale=0.568 | 0.216 | 0.937 |
|  | RC | Weibull | Shape=7.535, Scale=1.076 | 0.288 | 0.998 |
|  | OLSR | Normal | Mean=0.018, Sd=0.148 | 0.537 | 0.951 |
|  | MAR | Normal | Mean=-0.009, Sd=0.170 | 0.581 | 0.659 |
|  | RMAR | Normal | Mean=-0.001, Sd=0.165 | 0.362 | 0.740 |
|  | SMI | Logistic | Location=8.063, Scale=0.100 | 0.489 | 0.918 |

MLR - Mas/Length Ratio; BMI - Body Mass Index; FKI - Fulton’s K Index; RC - Relative Condition; OLSR – Ordinary Least Squares Regression Residuals; MAR – Major Axis Regression Residuals; RMAR – Reduced Major Axis Regression Residuals; SMI - Scaled Mass Index

**Table S3.** Distributions fitted to the observed data of biometric body condition indices (bBCI) by species of Southern European mammalian carnivores. The log-likelihood and Anderson-Darling tests were used to select the distributions that best fit the data, and Kolmogorov-Smirnov was used to test the hypothesis that the simulated and the observed data belong to the same distribution. Analysis performed using the package “fitdistrplus” [59] in R [58].

| **Species** | **Reference ranges** | **Physiological**  **parameter** | **Reference range sample size (n)** | **Data sample size (n)** | **Kolmogorov-Smirnov**  **posterior distribution (p)** |
| --- | --- | --- | --- | --- | --- |
| *Herpestes ichneumon* | [77] | Albumin | 0 | 22 | No reference data ^(1)^ |
|  |  | Globulins | 0 | 22 | No reference data ^(1)^ |
|  |  | Urea | 5 | 22 | 0.991 |
|  |  | Creatinine | 5 | 22 | 0.842 |
|  |  | Total bilirubin | 4 | 21 | 0.946 |
|  |  | Cholesterol | 4 | 22 | 0.948 |
|  |  | Triglycerides | 4 | 22 | 1.0 |
|  |  | Hemoglobin | 5 | 23 | 0.999 |
| *Vulpes vulpes* | [78] | Albumin | 226 | 23 | 0.082 |
|  |  | Globulins | 19 | 23 | 0.079 |
|  |  | Urea | 236 | 25 | <0.001 ^(2)^ |
|  |  | Creatinine | 228 | 24 | 0.441 |
|  |  | Total bilirubin | 19 | 23 | 0.355 |
|  |  | Cholesterol | 203 | 24 | 0.157 |
|  |  | Triglycerides | 77 | 24 | 0.109 |
|  |  | Hemoglobin | 247 | 11 | 0.008 ^(2)^ |
| *Genetta genetta* | [78,79] | Albumin | 37 | 4 | 0.991 |
|  |  | Globulins | 20 | 4 | 0.137 |
|  |  | Urea | 20 | 4 | 0.101 |
|  |  | Creatinine | 36 | 4 | 0.590 |
|  |  | Total bilirubin | 20 | 4 | 0.050 |
|  |  | Cholesterol | 20 | 4 | 0.989 |
|  |  | Triglycerides | 0 | 4 | No reference data ^(1)^ |
|  |  | Hemoglobin | 15 | 9 | 0.541 |
| *Martes foina* | [78] ^(3)^ | Albumin | 95 | 2 | 0.722 |
|  |  | Globulins | 92 | 2 | 0.719 |
|  |  | Urea | 103 | 2 | 0.042 ^(2)^ |
|  |  | Creatinine | 103 | 2 | 0.065 |
|  |  | Total bilirubin | 92 | 2 | 0.311 |
|  |  | Cholesterol | 86 | 2 | 0.740 |
|  |  | Triglycerides | 38 | 2 | 0.277 |
|  |  | Hemoglobin | 76 | 2 | 0.916 |
| *Canis lupus* | [78] | Albumin | 1546 | 15 | 0.012 ^(2)^ |
|  |  | Globulins | 1338 | 18 | 0.146 |
|  |  | Urea | 1641 | 19 | <0.001 ^(2)^ |
|  |  | Creatinine | 1641 | 17 | 0.091 |
|  |  | Total bilirubin | 1425 | 18 | 0.098 |
|  |  | Cholesterol | 1392 | 7 | 0.186 |
|  |  | Triglycerides | 558 | 7 | 0.107 |
|  |  | Hemoglobin | 1584 | 12 | 0.699 |
| *Felis silvestris* | [78-81] | Albumin | 216 | 5 | 0.222 |
|  |  | Globulins | 194 | 5 | 0.643 |
|  |  | Urea | 9 | 5 | 0.446 |
|  |  | Creatinine | 21 | 5 | 0.121 |
|  |  | Total bilirubin | 194 | 5 | 0.067 |
|  |  | Cholesterol | 178 | 5 | 0.565 |
|  |  | Triglycerides | 142 | 5 | 0.108 |
|  |  | Hemoglobin | 250 | 2 | 0.054 |

^(1)^ Reference ranges are not available. Only data was used to calculate the percentiles.

^(2)^ Reference values available do not belong to the same distribution as the data. Only data was used to calculate the percentiles.

^(3)^ Reference ranges for *Martes foina* are not available. Thus, the reference range for *Martes pennant* was used.

**Table S4**. Summary of the data used to estimate the posterior distribution of the physiological parameters by species of Southern European mammalian carnivores. The posterior distribution merged, in a probability tree, the published reference ranges and the observed data and was compared with the later using the Kolmogorov-Smirnov test. The complete dataset of hematology and serum biochemistry parameters was available for 42 animals, but data on individual parameters is available for a larger number of animals. Analysis performed using the package ‘“mc2d” [62] in R [58].

| **Species** | | **MLR** | **BMI** | **FKI** | **RC** | **OLSR** | **MAR** | **RMAR** | **SMI** |
| --- | --- | --- | --- | --- | --- | --- | --- | --- | --- |
| *Vulpes vulpes* | Mean ± SD | 4.819 ± 1.165 | 4.898 ± 0.868 | 5.121 ± 1.269 | 1.014 ± 0.178 | 0.000 ± 0.165 | 0.000 ± 0.175 | 0.000 ± 0.168 | 8.410 ± 0.209 |
|  | Min – Max | 2.165 - 7.740 | 3.426 - 8.533 | 3.239 - 11.378 | 0.718 - 1.878 | -0.331 - 0.630 | -0.359 - 0.716 | -0.334 - 0.680 | 8.000 - 9.284 |
| *Herpestes ichneumon* | Mean ± SD | 2.145 ± 0.541 | 2.456 ± 0.450 | 2.863 ± 0.561 | 1.014 ± 0.177 | 0.000 ± 0.167 | 0.000 ± 0.180 | 0.000 ± 0.171 | 7.478 ± 0.188 |
|  | Min - Max | 1.014 - 3.368 | 1.470 - 3.747 | 1.857 - 5.890 | 0.690 - 1.735 | -0.370 - 0.551 | -0.431 - 0.769 | -0.390 - 0.679 | 7.055 - 8.356 |
| *Genetta genetta* | Mean ± SD | 1.534 ± 0.372 | 1.767 ± 0.301 | 2.063 ± 0.254 | 1.007 ± 0.124 | 0.000 ± 0.119 | 0.000 ± 0.123 | 0.000 ± 0.120 | 7.127 ± 0.125 |
|  | Min – Max | 0.539 - 2.232 | 0.967 - 2.420 | 1.581 - 2.796 | 0.787 - 1.445 | -0.240 - 0.368 | -0.221 - 0.490 | -0.216 - 0.437 | 6.912 - 7.637 |
| *Martes foina* | Mean ± SD | 1.846 ± 0.302 | 2.714 ± 0.360 | 4.019 ± 0.585 | 1.009 ± 0.132 | 0.000 ± 0.132 | 0.000 ± 0.155 | 0.000 ± 0.140 | 7.115 ± 0.146 |
|  | Min - Max | 1.064 - 2.453 | 1.981 - 3.472 | 2.770 - 5.877 | 0.739 - 1.296 | -0.303 - 0.260 | -0.381 - 0.472 | -0.347 - 0.329 | 6.761 - 7.527 |
| *Canis lupus* | Mean ± SD | 19.083 ± 4.164 | 12.833 ± 2.040 | 8.741 ± 1.409 | 1.012 ± 0.150 | 0.000 ± 0.155 | 0.000 ± 0.170 | 0.000 ± 0.160 | 10.213 ± 0.239 |
|  | Min - Max | 8.929 - 25.455 | 7.846 - 16.390 | 5.812 - 13.287 | 0.652 - 1.339 | -0.427 - 0.292 | -0.379 - 0.503 | -0.400 - 0.411 | 9.764 - 11.093 |
| *Felis silvestris* | Mean ± SD | 4.089 ± 0.985 | 4.937 ± 0.873 | 6.037 ± 1.067 | 1.015 ± 0.170 | 0.000 ± 0.177 | 0.000 ± 0.199 | 0.000 ± 0.185 | 8.081 ± 0.220 |
|  | Min - Max | 2.143 - 5.742 | 3.061 - 6.662 | 3.906 - 8.385 | 0.653 - 1.321 | -0.426 - 0.279 | -0.410 - 0.459 | -0.417 - 0.379 | 7.673 - 8.626 |

MLR - Mas/Length Ratio; BMI - Body Mass Index; FKI - Fulton’s K Index; RC - Relative Condition; OLSR – Ordinary Least Squares Regression Residuals; MAR – Major Axis Regression Residuals; RMAR – Reduced Major Axis Regression Residuals; SMI - Scaled Mass Index

**Table S5.** Descriptive statistics of the observed biometric body condition indices by species of Southern European mammalian carnivores.

A)


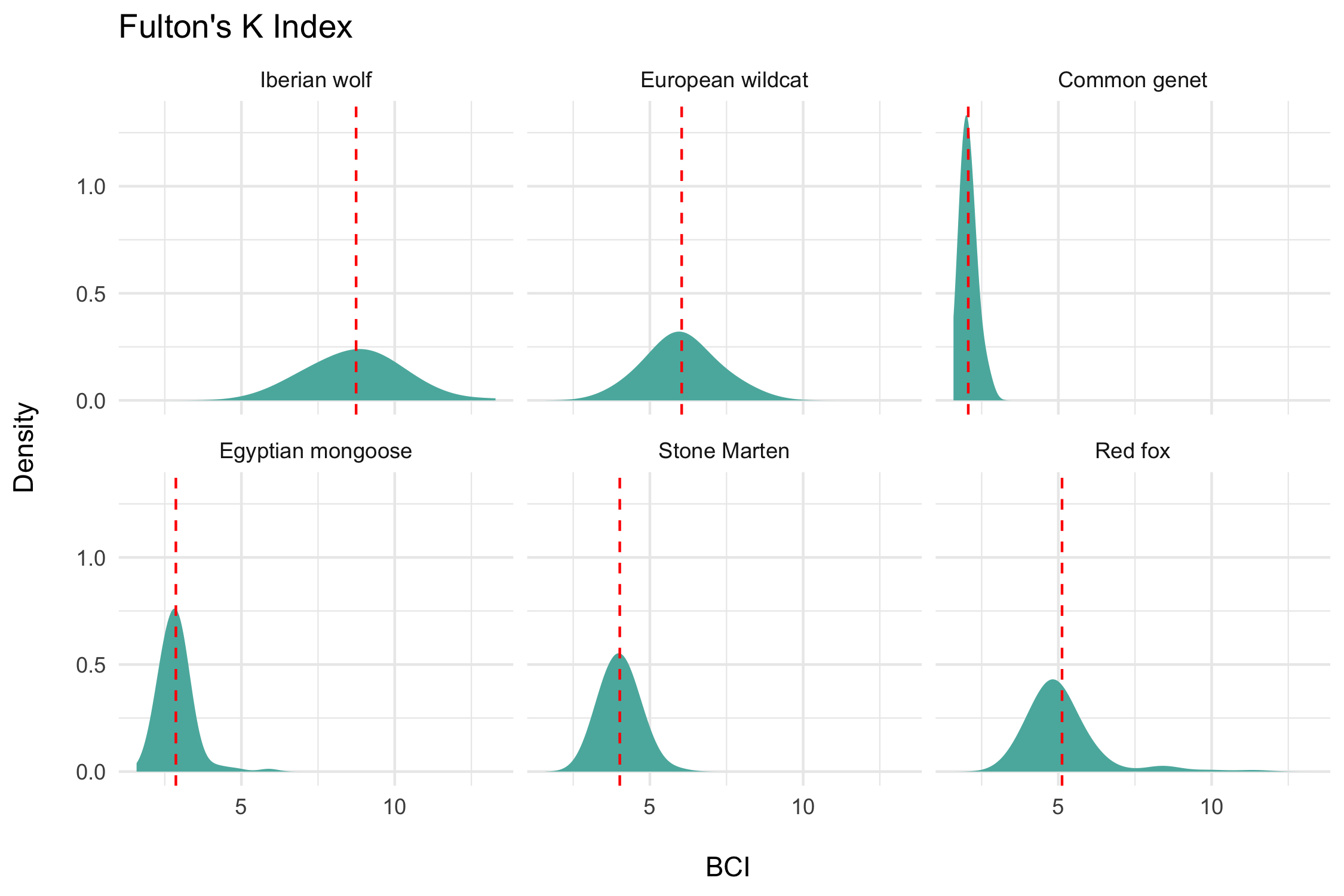


B)


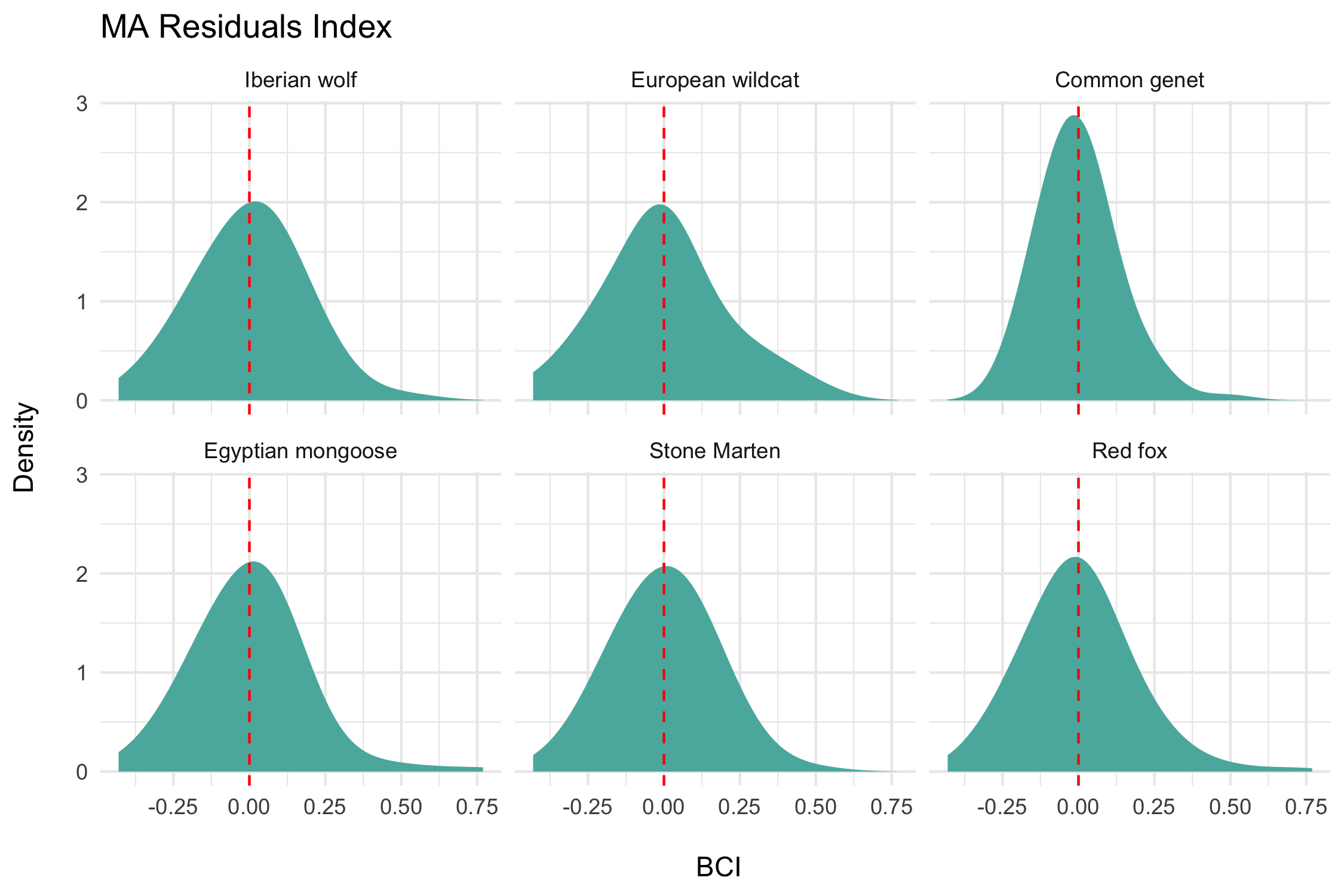


C)


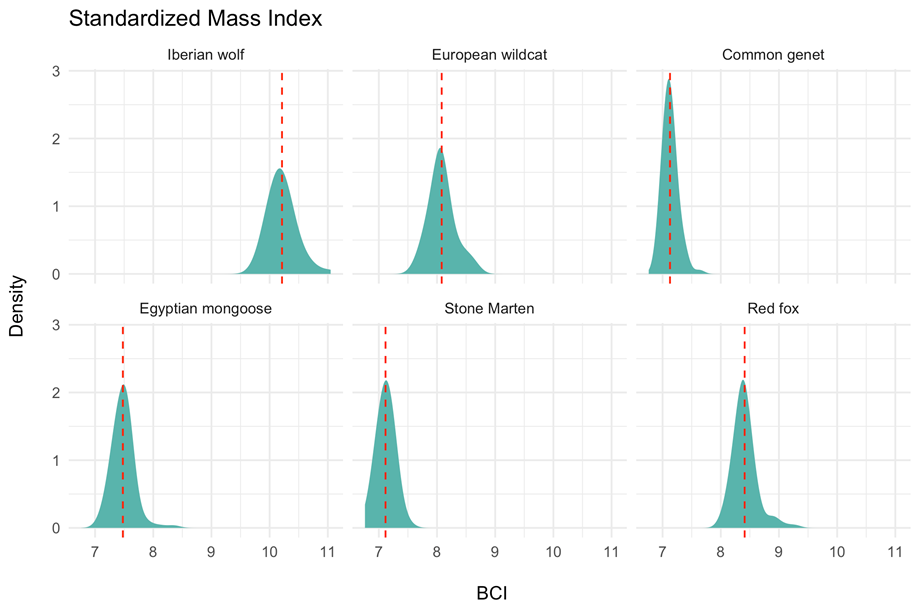


**Figure S3.** Species-specific distributions fitted to the observed data of the three selected biometric body condition indices. Fulton’s K Index (A),

Major Axis Regression Residuals (B) and Scaled Mass Index (C), with respective mean (dashed red vertical line).

**References Supplementary Information**

77. Palomares, F., Delibes, M., Recio, F. Hematology and serum biochemistry of the Egyptian mongoose, *Herpestes ichneumon*. J Wildl Dis **28**(4), 659-661 (1992).

78. ZIMS Expected Test Results for Carnivores. Species360 Zoological Information Management System. Retrieved from <http://zims.Species360.org>. Accessed 11^th^ April 2018.

79. Millán, J., Chirife, A. D., Altet, L. Serum chemistry reference values for the common genet (*Genetta genetta*), variations associated with *Leishmania infantum* infection. Vet. Quart. **35**(1), 43-47 (2015).

80. Račnik, J. et al. (2004) Blood and urine values of free-living European wildcats in Slovenia. Eur. J. Wildl. Res. **50**(1), 44-47.

81. Marco, I., Martinez, F., Pastor, J., Lavin, S. Hematologic and serum chemistry values of the captive European wildcat. J. Wildl. Dis. **36**(3), 445-449 (2000).
